# PARP Inhibitors: Clinical Relevance, Mechanisms of Action and Tumor Resistance

**DOI:** 10.3389/fcell.2020.564601

**Published:** 2020-09-09

**Authors:** Maddison Rose, Joshua T. Burgess, Kenneth O’Byrne, Derek J. Richard, Emma Bolderson

**Affiliations:** ^1^Cancer & Ageing Research Program, School of Biomedical Sciences, Institute of Health and Biomedical Innovation, Translational Research Institute, Queensland University of Technology, Brisbane, QLD, Australia; ^2^Princess Alexandra Hospital, Brisbane, QLD, Australia

**Keywords:** BRCA, PARP inhibitors, DNA damage, DNA repair, cancer, targeted therapy

## Abstract

The Poly (ADP-ribose) polymerase (PARP) family has many essential functions in cellular processes, including the regulation of transcription, apoptosis and the DNA damage response. PARP1 possesses Poly (ADP-ribose) activity and when activated by DNA damage, adds branched PAR chains to facilitate the recruitment of other repair proteins to promote the repair of DNA single-strand breaks. PARP inhibitors (PARPi) were the first approved cancer drugs that specifically targeted the DNA damage response in BRCA1/2 mutated breast and ovarian cancers. Since then, there has been significant advances in our understanding of the mechanisms behind sensitization of tumors to PARP inhibitors and expansion of the use of PARPi to treat several other cancer types. Here, we review the recent advances in the proposed mechanisms of action of PARPi, biomarkers of the tumor response to PARPi, clinical advances in PARPi therapy, including the potential of combination therapies and mechanisms of tumor resistance.

## Introduction

Cancer is a large subset of diseases characterized by the uncontrollable growth of abnormal cells. Globally, there are 17 million new cancer diagnoses each year, with an estimated 9.6 million cancer-related deaths occurring in 2018, placing an enormous burden on health care systems (Bray et al., 2018). The advances in targeted cancer therapies have gained significant momentum in recent years, although chemotherapy treatment regimens remain the gold standard in the treatment of several cancer types. Chemotherapeutic agents are designed to target rapidly dividing cells; however, the major disadvantage of this treatment type is that the drugs are unable to discriminate between malignant and non-malignant cells. Therefore, chemotherapy patients often experience off-target toxicity and detrimental side effects due to the impact of chemotherapy on healthy tissues. The most commonly experienced side effects are nausea and vomiting, with greater than 90% of chemotherapy patients requiring anti-emetic medications whilst undergoing treatment (Lorusso et al., 2017). Additional patient reported side effects include fatigue, generalized pain and other gastrointestinal disturbances (Pearce et al., 2017). In contrast, targeted therapies directly target cancer-specific mutations and abnormalities to inhibit tumor growth and progression, while minimizing the effects on surrounding non-malignant tissue. Targeted therapies are often associated with more favorable patient outcomes, given they are significantly less likely to result in off-target side effects.

PARP Poly (ADP-ribose) polymerases are a family of 17 proteins involved in several cellular processes, including the stress response, chromatin remodeling, DNA repair and apoptosis (Krishnakumar and Kraus, 2010; Pines et al., 2012; Hu et al., 2014; Zhao Q. et al., 2019). The most well recognized and characterized member of the PARP protein family is PARP1, initially identified for its role in the detection and repair of single-strand DNA breaks (Fisher et al., 2007; Hanzlikova et al., 2016; Heeke et al., 2018). More recent evidence suggests that PARP1 may also have a role in alternative DNA repair pathways, including nucleotide excision repair, non-homologous end joining (both classical and alternative), homologous recombination and DNA mismatch repair (Wang et al., 2006; Haince et al., 2008; Sugimura et al., 2008; Bryant et al., 2009; Boehler et al., 2011; Rulten et al., 2011; Pines et al., 2012; Fenton et al., 2013; Min W. et al., 2013; Beck et al., 2014).

The first member of the PARP protein family was discovered in 1963 during investigations of an enzyme that was activated by nicotinamide mononucleotide (NMN) in a DNA dependent manner and hypothesized to have involvement in a PolyA producing reaction (Chambon et al., 1963). However, later studies revealed that the resulting molecule did not possess PolyA characteristics, given it had the adenylic moiety of ATP and the ribose and phosphate moieties of NMN. Thereby, suggesting the enzyme had transglycosidase activity which catalyzes the polymerization of nicotinamide adenine dinucleotide (NAD) intermediates to form an ADP-ribose polymer, via the simultaneous formation of ribose-ribose bonds and removal of the nicotinamide residues (Chambon et al., 1969). In 1967, numerous studies further identified and characterized this ADP-ribose polymer producing enzyme (Fujimura et al., 1967; Hasegawa et al., 1967; Nishizuka et al., 1967; Reeder et al., 1967; Sugimura et al., 1967). Reeder et al. (1967) and Sugimura et al. (1967) independently identified the reactant product as the negatively charged polymer termed poly(ADP-ribose) (PAR).

Poly (ADP-ribose) polymerase (PARP) inhibitors (PARPi) are a novel class of anti-cancer therapies which compete with NAD^+^ for the catalytically active site of PARP molecules. PARPi have shown to be effective in the treatment of homologous recombination repair (HR) deficient tumors. Specifically, PARP inhibitors have been used to target tumors with mutations in the essential HR genes, Breast Cancer Associated 1 and 2 (BRCA1 and BRCA2) (Fong et al., 2009, 2010; Coleman et al., 2019; Tuli et al., 2019). Several PARP inhibitors have been approved for the treatment of BRCA-mutated ovarian, breast and pancreatic cancer. In addition, there are currently 269 clinical trials registered on clinicaltrials.gov examining the use of PARP inhibitors as an anti-cancer therapy in chemo-resistant germline or somatic BRCA1/2 mutated breast, ovarian, lung, and pancreatic cancers (Dockery et al., 2017).

## PARP1 and Single-Strand Break Repair (SSBR)

PARP1 is vital for the repair of single-strand breaks (Fisher et al., 2007; Hanzlikova et al., 2016). Since single-strand breaks are also produced as an intermediate of Base-Excision Repair (BER); PARP is also sometimes considered to be required for BER, as suggested by several studies (Dantzer et al., 1999, 2000). However, there is contradictory evidence for the sensitivity of PARP1 deficient or PARP1 inhibited cells to agents that induce base damage (de Murcia et al., 1997; Dantzer et al., 1999; Vodenicharov et al., 2000; Allinson et al., 2003; Pachkowski et al., 2009). Another study found that PARP was not required to repair base damage but was required to repair hydrogen peroxide-induced single-strand breaks (Strom et al., 2011). There is also some evidence that PARP1 dependent and independent pathways of SSBR may exist with one study showing that PARP1 was required for SSBR in G1 but not S phase of the cell cycle. In contrast PARPi inhibited SSBR in all phases of the cell cycle (Godon et al., 2008).

**FIGURE 1:**
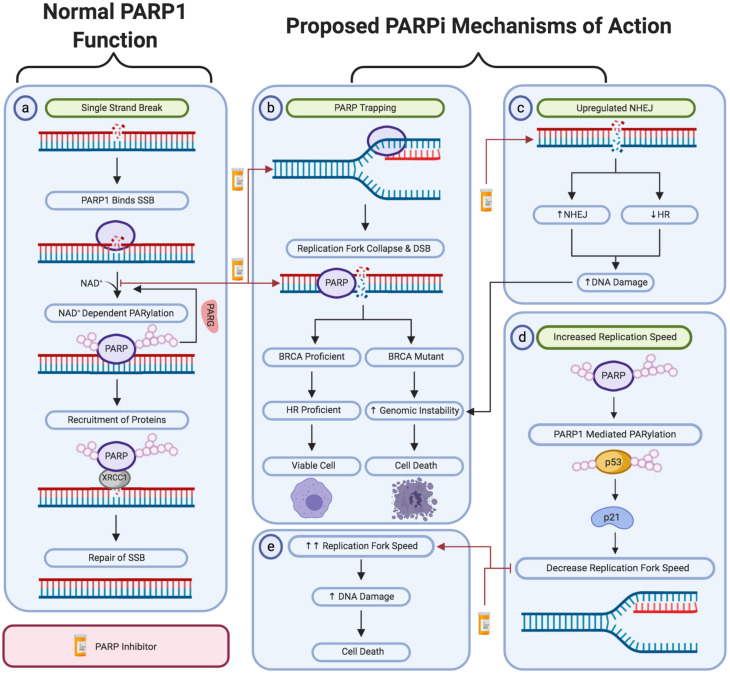
A schematic representation of PARP1 activity in single-strand break repair and the proposed mechanism of action of PARP inhibitors. **(a)** The activity of PARP1 in the repair of oxidative stress-induced single-strand breaks via the base excision repair pathway. The proposed PARPi-induced: **(b)** PARP trapping mechanism. **(c)** Upregulation of non-homologous end joining activity and downregulation of homologous recombination repair. **(d,e)** Loss of negative regulation of replication fork speed. Created with Biorender.

DNA damage is rapidly detected through the conserved N-terminal DNA-damage sensing and binding domain of PARP (Ali et al., 2012). Subsequently, PARP1 catalyzes the post-translational polymerization of ADP-ribose units (PARs) from NAD^+^ molecules onto target proteins via covalent linkages to acidic residues (Bian et al., 2019). PARP1 activation enables the auto-PARylation of PARP1 itself at serine, tyrosine and glutamic acid residues within the PARP1 auto-modification domain. This auto-PARylation further activates PARP1 and enables the PARylation of histones and other chromatin-associated proteins (Chaudhuri and Nussenzweig, 2017). Collectively, this auto- and hetero-modification recruits additional DNA repair molecules, such as XRCC1 to the site of damage, promoting the effective repair of DNA (Figure 1a) (Liu et al., 2017).

PARP2 and PARP3 also have roles in DNA repair processes and share partial redundancy with PARP1 in some of these roles. Demonstrating this redundancy, PARP2 deficient mice display post-replicative genomic instability and PARP1 and PARP2 double mutant mice are embryonic lethal (Ménissier de Murcia et al., 2003). PARP2 also has a role in SSBR and has an overlapping role with PARP1 for recruitment of XRCC1 (Hanzlikova et al., 2017). In addition, PARP3 deficient cells also display genome instability and delayed repair of single-strand breaks, but no radiosensitivity (Boehler et al., 2011). PARP1, PARP2, and PARP3 share structural similarities and were also shown to be activated in a similar manner through DNA-dependent catalytic activation through a local destabilization of the catalytic domain (Langelier et al., 2014).

## DNA Double-Strand Break Repair Pathways

Targeted therapies, such as PARPi, have greater specificity and less off-target side effects than traditional therapies, such as chemotherapy or radiation treatment, and can lead to more favorable outcomes in cancer patients. As mentioned previously, PARPi have been found to target tumors with defects in the HR pathway due to BRCA1 or BRCA2 mutations but have little toxicity on normal cells with functional HR. The two main pathways of DNA double strand break (DSB) repair are briefly described below.

### Homologous Recombination Repair

Although HR is considered the least error-prone form of DSB repair, it is restricted to the S and G2 phases of the cell cycle due to the requirement of a template sister chromatid (Brandsma and Gent, 2012).

HR is a complex process, requiring a myriad of proteins. The MRN-complex, composed of MRE11, Rad50 and Nbs1 has several roles in the DNA damage response. Most well recognized, is the role of the MRN-complex as a sensor of DSB to initiate HR following their detection (Krajewska et al., 2015). The MRN-complex is rapidly recruited to the sites of DSBs, facilitating the recruitment and full activation of the ATM kinase and initiates the subsequent ATM-mediated phosphorylation of each member of the MRN-complex. This then promotes further recruitment of the MRN-complex and initiates ATM-dependent downstream signaling (Cassani et al., 2019). The MRN-complex, in conjunction with CtIP, then initiates the 5′ to 3′ nucleolytic resection of the DNA to produce a 3′ overhang of single-stranded DNA (Zhu et al., 2008; Yun and Hiom, 2009; Brandsma and Gent, 2012). This end strand resection is further mediated by other exonuclease proteins, such as Exo1.

The resulting 3′ overhang is then bound by a RPA heterotrimer at a high affinity, mediating the removal of a secondary structure and protecting the section of ssDNA (Chen et al., 2013). Subsequently, the BRCA1 and BRCA2-mediated displacement of RPA by Rad51 occurs, forming a helical nucleoprotein filament on the single-stranded DNA (Jensen et al., 2010). This filament locates a homologous sequence of DNA and catalyzes strand invasion to form a Holliday junction intermediate (Hiom, 2001). The 3′ end of the invading strand is then used to prime DNA synthesis and extend the region of homology. The resulting Holliday junction is resolved, primarily by the BTR complex, consisting of Bloom’s syndrome helicase (BLM), topoisomerase IIIα, RMI1, and RMI2 (Xue et al., 2013). Holliday junction dissolution signals the completion of HR activity, indicating the effective repair of the dsDNA break (Matos and West, 2014; Ma et al., 2017).

### Non-homologous End Joining

Unlike HR, non-homologous end joining (NHEJ) does not require a homologous template for the repair of DSBs and directly ligates DNA ends (Khanna and Jackson, 2001; Davis and Chen, 2013). Furthermore, it is active throughout all phases of the cell cycle (Mao et al., 2008).

Given the lack of a template strand, NHEJ is considered to be a comparatively error prone DSB repair mechanism, associated with an increased prevalence of nucleotide insertions and deletions and therefore, a greater probability for genomic instability (Bassing and Alt, 2004). NHEJ is initiated by the binding of a Ku heterodimer, composed of the Ku70 and Ku80 proteins, to a DSB (Sishc and Davis, 2017). The Ku70/80 heterodimer then acts as a scaffold protein to recruit and activate DNA-dependent protein kinase (DNA-PKcs) at the site of damage and produce a catalytically active complex. DNA-PKcs mediated bridging across the break enables DNA resection or gap-filling by several known enzymes. The Ligase IV/XRCC4 complex then ligates the DNA ends back together (Sharma et al., 2016).

## PARP Inhibitors – Synthetic Lethality

Poly (ADP-ribose) polymerases (PARP) inhibitors (PARPi) are a class of anti-cancer drugs which compete with nicotinamide (NAD^+^) for the catalytically active site of PARP molecules. Inhibition of PARP activity was initially demonstrated in 1971, following treatment of HeLa cells with thymidine and nicotinamide (Preiss et al., 1971). Several later studies identified numerous benzamides as inhibiting PARP activity via NAD^+^ competition. However, these compounds were considered clinically unviable due to their low potency and specificity (Purnell and Whish, 1980; Canan Koch et al., 2002; Skalitzky et al., 2003). Although PARP1 is generally considered the major target of PARPi, due to the structural similarity of the NAD-binding domain of some of the PARP family members, some PARPi also inhibit the activity of other PARPs, including PARP2 and PARP3 and some other off-target effects on kinases have also been observed (Murai et al., 2012b; Antolin et al., 2020).

PARPi have been shown to be effective against homologous recombination repair deficient tumors in a synthetically lethal interaction. Synthetic lethality is where loss of one gene is compatible with cell viability; however, simultaneous disruption of two genes results in cell death (Geenen et al., 2018). The synthetic lethality between PARP inhibition and BRCA mutation or depletion was first observed in 2005, where it was originally hypothesized that inhibition of PARP1 activity would lead to replication fork collapse and the subsequent HR-dependent repair of these forks. Therefore, given that BRCA1/2 mutated tumor cells have disrupted HR activity, the collapsed replication forks are unable to be repaired and cell death occurs (Bryant et al., 2005; Farmer et al., 2005).

There are currently several PARP inhibitors approved for the treatment of BRCA1/2 mutated breast, ovarian, pancreatic and prostate cancers. Due to the relatively low frequency of BRCA1/2 mutations, this limits their applicability to the treatment of 10–15% of breast and ovarian tumors, 4–7% of pancreatic tumors and 1.5% of prostate carcinoma (Bryant et al., 2005; Iqbal et al., 2012; Oh et al., 2019). However, more recent studies suggest that PARP inhibitors may have much wider applications. This includes the treatment of tumors with alternative HR deficiencies or mutations in other DNA damage response genes (Bryant et al., 2005; Turner et al., 2008; Jonsson et al., 2019). Tumors with high levels of oxidative and replicative stress may also be sensitive to PARP inhibitors as a monotherapy, irrespective of HR status (Majuelos-Melguizo et al., 2015; Kukolj et al., 2017; Schoonen et al., 2017; Michelena et al., 2018).

The indications for which PARP inhibitors have been approved for are summarized below (Table 1). In 2014, Olaparib (Lynparza) was the first PARPi approved by the Food and Drug Agency (FDA) and European Medicines Agency (EMA) as a monotherapy for the treatment of advanced, germline BRCA mutated ovarian cancer (Kaufman et al., 2015). In 2017, this was extended to include maintenance therapy of reoccurring ovarian, fallopian and primary peritoneal tumors, regardless of BRCA mutational status (Pujade-Lauraine et al., 2017; Friedlander et al., 2018). Olaparib has also been approved for the treatment of germline BRCA1/2 mutated HER2-negative breast and metastatic pancreatic cancer in 2018 and 2019, respectively (Moore et al., 2018; Golan et al., 2019; Robson et al., 2019). Most recently, Olaparib was approved for the treatment of HRD-positive metastatic castration-resistant prostate cancer (de Bono et al., 2020).

**TABLE 1:** Food and Drug Administration (FDA) and European Medicines Agency (EMA) approval history of PARP inhibitors.

PARP inhibitor	Approving organization	Year of approval	Indication	Mutational requirement	Relevant studies
Olaparib	FDA and EMA	2014	Advanced ovarian carcinoma	Germline BRCA1/2 Mutation	NCT0107662 (Kaufman et al., 2015)
	FDA and EMA	2017	Reoccurring ovarian, fallopian and primary peritoneal carcinoma	Independent of BRCA1/2 Mutational Status	SOLO-2 (Pujade-Lauraine et al., 2017) and Study 19 (Friedlander et al., 2018)
	FDA EMA	2018 2019	HER-2 negative breast cancer	BRCA1/2 Mutated	OlympiAD (Robson et al., 2017)
	FDA EMA	2018 2019	First-line treatment of advanced ovarian, fallopian and primary peritoneal carcinoma	Germline BRCA1/2 Mutation Complete or partial chemotherapy response.	SOLO-1 (Moore et al., 2018)
	FDA	2019	Metastatic pancreatic cancer	BRCA1/2 Mutated	POLO (Golan et al., 2019)
	FDA	2020	First-line treatment of advanced ovarian, fallopian and primary peritoneal carcinoma in combination with Bevacizumab	HRD-Positive Complete or partial chemotherapy response.	PAOLA-1 (Ray-Coquard et al., 2019)
	FDA	2020	Metastatic castration-resistant prostate cancer	HRD-positive	PROfound (de Bono et al., 2020)
Rucaparib	FDA EMA	2016 2018	Advanced ovarian carcinomas, following multiple chemotherapy treatments	BRCA1/2 Mutated	ARIEL2 and Study 10 (Oza et al., 2017)
	FDA EMA	2018 2019	Reoccurring ovarian, fallopian and primary peritoneal carcinoma	Independent of BRCA1/2 Mutational Status	ARIEL3 (Coleman et al., 2017)
	FDA	2020	Metastatic castration-resistant prostate cancer	BRCA1/2 Mutated	TRITON2 (Abida et al., 2019)
Niraparib	FDA and EMA	2017	Reoccurring ovarian, fallopian and primary peritoneal carcinoma	Complete or partial chemotherapy response.	ENGOT-OV16/NOVA Study (Mirza et al., 2016)
	FDA	2019	Reoccurring ovarian, fallopian and primary peritoneal carcinoma	HRD-positive Independent of chemotherapy response	QUADRA Study (Moore et al., 2019)
	FDA and EMA	2020	Advanced ovarian carcinomas and primary peritoneal carcinoma	Independent of biomarker status Complete or partial chemotherapy response.	PRIMA Study (Gonzalez-Martin et al., 2019)
Talazoparib	FDA and EMA	2018	Advanced or metastatic HER2-negative breast cancer	Germline BRCA1/2 Mutated	EMBRACA Study (Ettl et al., 2018)

Several other PARP inhibitors, including Rucaparib (Rubraca), Niraparib (Zejula), and Talazoparib (Talzenna) have also been approved for use in various clinical settings. In 2016, Rucaparib was granted an accelerated approval for the treatment of germline or somatic BRCA1/2-mutated advanced ovarian carcinomas, following multiple chemotherapy treatments (Oza et al., 2017). Subsequently, Rucaparib maintenance therapy was approved in 2018 for reoccurring ovarian, fallopian and primary peritoneal, regardless of BRCA mutational status (Coleman et al., 2017). In May 2020, Rucaparib gained FDA approval for the treatment BRCA1/2 mutated metastatic castration-resistant prostate cancer (Abida et al., 2019).

Niraparib was initially approved in 2017 for the maintenance treatment of reoccurring ovarian, fallopian and primary peritoneal carcinomas, regardless of BRCA mutational status that show a complete or partial chemotherapy response (Mirza et al., 2016). In 2019, this was expanded to the late-line treatment of the aforementioned carcinomas, that were specifically HRD-positive, irrespective of prior sensitivity to chemotherapy (Moore et al., 2019). Subsequently, this was further expanded in 2020 to include the treatment of reoccurring ovarian, fallopian and primary peritoneal carcinomas that have previously shown complete or partial response to chemotherapy, independent of biomarker status (Gonzalez-Martin et al., 2019).

In 2018, Talazoparib was approved for the treatment of germline BRCA1/2-mutated advanced or metastatic HER2-negative breast cancer (Ettl et al., 2018). Since this approval, Talazoparib has not gained approval for the treatment of any further malignancies.

A fifth PARPi, Veliparib (ABT-888) is currently undergoing clinical trials; however, is not yet approved for use in clinical practice (Kummar et al., 2009; Baxter et al., 2020). Lastly, Fluzoparib (HS10160) was initially identified in 2017 as a novel PARPi (Jhan and Andrechek, 2017). Clinical trials for Fluzoparib commenced in 2019 for the treatment of solid tumors, including ovarian, breast, pancreatic and lung cancer (Han et al., 2019; Luo et al., 2019).

## PARPi Biomarkers

Biomarkers which can predict the PARPi sensitivity of tumors are of great interest within the scientific community. The identification of biomarkers will not only further our understanding of the mechanism by which PARP inhibitors mediate their anti-cancer capacity but may also increase the subset of patients treated with PARP inhibitors. Since their approval in 2014, significant efforts have been made to establish validated biomarkers for PARPi sensitivity, but with little success. As such, germline and somatic BRCA1/2 mutations remain the main predictive biomarkers for the majority of PARP inhibitors (Ganguly et al., 2016). However, in 2019, a Homologous Recombination Deficiency (HRD) assay was approved as biomarker for the use of Niraparib in patients with advanced ovarian cancer.

### The BRCA1 and BRCA2 Genes

The Breast Cancer Susceptibility Genes, BRCA1 and BRCA2, have well established roles in the maintenance of genomic stability. Germline mutations in the tumor suppressor BRCA1 and BRCA2 genes have been strongly associated with an increased risk of breast and ovarian cancer (Antoniou et al., 2003; Brekelmans et al., 2006). Specifically, it is estimated that a woman’s lifetime risk of developing breast or ovarian cancer without a BRCA mutation is approximately 12 and 1.3%, respectively (Kotsopoulos, 2018; Pasanisi and Bruno, 2018). However, in women carrying a harmful BRCA1 mutation this is elevated to 60% lifetime risk of developing breast cancer and 44% risk of developing ovarian cancer (Cavanagh and Rogers, 2015). Similarly, it is estimated that women carrying harmful BRCA2 mutations have a 26 and 17% lifetime risk of inheriting breast and ovarian cancer, respectively (Kuchenbaecker et al., 2017). These mutations are of substantial prevalence, with between 1/400 and 1/800 people carrying a harmful BRCA1/2 mutation (Hall et al., 2009).

Collectively, more than 3500 pathogenic BRCA1/2 mutations have been identified (Godet and Gilkes, 2017). Many of the BRCA1 mutations are frame shift mutations which have a deleterious effect on BRCA1 protein expression, resulting in a non-functional or missense protein. In individuals that have inherited a single mutated BRCA1/2 allele, the wild-type allele is often somatically mutated or silenced as they age (Godet and Gilkes, 2017). This second event often leaves the individual without a functional BRCA1/2 allele and significantly increases the mutation burden within their cells (Petrucelli et al., 2010). BRCA2 frame shift mutations have been shown to frequently result in premature truncation of proteins. Many of these mutations render the BRCA2 gene ineffective and the cells are unable to perform HR repair of stalled replication forks or DSBs.

Under current guidelines, women presenting with breast or ovarian tumors are routinely tested for hereditary mutations in BRCA1/2 and this guides whether they are treated with PARP inhibitors. A recent study showed that over 40% of BRCA1/2 mutations were somatic, suggesting that the tumors should also be tested, to identify more patients that would benefit from PARP inhibitor treatment (Vos et al., 2020.) However, growing evidence suggests that BRCA1/2 mutational status does not always accurately correlate with PARPi sensitivity (Jonsson et al., 2019) and there is a need to find more accurate predictive PARPi biomarkers.

A recent study of ovarian cancer samples, from patients treated with Olaparib maintenance therapy, indicated that Olaparib also significantly improved survival outcomes in patients who lacked BRCA1/2 mutations; but harbored other DDR gene mutations. This indicates that alternative DDR proteins, beyond BRCA1/2, may have the capacity to be an effective PARPi biomarker (Hodgson et al., 2018). Several HR repair mutations have been identified as potential prospective PARPi biomarkers, including ATM, FANC A/F, CHK2, RAD51B/C and CDK12 (Mateo et al., 2015; Criscuolo et al., 2019).

### Homologous Recombination Deficiency Score

Homologous recombination deficiency score is defined as the unweighted sum of the loss of heterozygosity (LOH) score, telomeric-allelic imbalance (TAI) score and large-scale state transitions (LST) score. HRD score has been previously identified as a predictive biomarker for tumor response to neoadjuvant chemotherapy treatment (Telli et al., 2016). Tumors with BRCA1/2 mutations are recognized to have the highest HRD scores; however, tumors with homologous recombination repair defects have also been shown to have intermediate HRD scores (Hodgson et al., 2018). Given tumors with HR deficiencies have been shown to be more sensitive to PARP inhibitors than HR proficient tumors, it was hypothesized that HRD score may be an effective PARPi biomarker. However, studies have shown mixed outcomes about the applicability of HRD score as a PARPi biomarker. Several studies have been conducted examining the link between HRD score and Progression Free Survival (PFS) in BRCA wild-type tumors. PFS is defined as the period of time in which a tumor does not worsen following a treatment regime. Hurley et al. (2019) showed that higher HRD scores did correlate with significantly greater PFS following Niraparib treatment in BRCA wild-type ovarian cancer. However, an earlier study indicated that HRD status did not strongly correlate with tumor shrinkage following Veliparib treatment (Mirza et al., 2016; Hurley et al., 2019).

Furthermore, several observational studies have been conducted to investigate potential predictive biomarkers of PARPi response; however, significant research is required to validate these targets prior to them being implicated in clinical practice. These include biomarkers other than gene mutations, including hypermethylation of the promoter regions of BRCA1 and RAD51, hypermethylation of H0XA9 in circulating DNA, high expression of Ku80 and low 53BP1 expression (Montavon et al., 2012; Kondrashova et al., 2018).

## Proposed Mechanisms of Action of PARP Inhibitors

The underlying mechanism of action by which PARP inhibitors induce their anti-cancer activities has yet to be fully uncovered. However, recent findings have significantly improved our understanding of PARPi activity, and several broadly recognized theories have emerged, although a consensus is yet to be reached.

### Inhibiting Single Strand Break Repair

PARP1 has been identified to have an essential role in Single-Strand Break Repair (SSBR). Therefore, it was initially hypothesized that PARP inhibitors may induce lethality by impairing the repair of DNA single-strand breaks and leading to the accumulation of damage (Bryant et al., 2005). However, other studies suggest that the synthetic lethality induced by PARP inhibitors is not due to the inhibition of SSBR. Supporting this, there is little evidence that PARP inhibitors lead to the accumulation of DNA single-strand breaks (Gottipati et al., 2010). In addition, siRNA-mediated depletion of XRCC1, a key protein in the SSBR response, did not increase sensitivity to PARP depletion via PARP1 siRNA (Nazarkina et al., 2007; Patel et al., 2011). Although, XRCC1 depletion did increase the sensitivity to two PARP inhibitors, Olaparib and Veliparib, in cellular cytotoxic assays (Horton et al., 2014). This is consistent with findings that genetically inhibiting PARP is significantly less cytotoxic than utilizing a PARPi, which may be expected to be similarly cytotoxic if the mechanism of PARPi toxicity was due to inhibiting SSBR (Murai et al., 2012a). In light of the above, this suggested that PARPi sensitivity may be mediated via other mechanisms in addition to inhibiting SSBR.

### Replication Fork Stalling and PARP Trapping

It is well recognized that PARP activation is required at the site of stalled replication forks to facilitate MRE11-mediated restart of replication (Bryant et al., 2009; Koppensteiner et al., 2014). DNA DSBs are likely to arise following the collision of the replication fork with a DNA lesion or single strand break (Liao et al., 2018). Based on these findings, it was hypothesized that PARP inhibitors may induce tumor cell death because stalled replication forks are unable to be restarted in PARP inhibited homologous recombination repair-deficient cells. This is supported by the evidence that PARP inhibitors are synthetically lethal with tumors which possess either HR or fork stabilization defects (Liao et al., 2018).

The PARP trapping mechanism of PARP inhibitors is also linked to replication fork stalling and is one of the most well-established theories. This proposed mechanism also offers insight into why inhibiting PARP activity is significantly more cytotoxic than genetically removing PARP1 through methods such as small-interfering RNA (siRNA) technologies (Murai et al., 2012a). The initial PARP trapping theory proposed that PARP inhibitors competitively bind to the NAD^+^ binding domain on PARP1. This results in PARP1 becoming trapped on the DNA due to the inability to auto-PARylate PARP1 (Shen et al., 2013). There is strong evidence supporting this theory, including the observation that PARP1-DNA complexes pre-exposed to a PARPi had less ability to dissociate following NAD^+^ induced auto-modification of PARP1. Therefore, indicating that the PARPi mechanism could involve PARP trapping to some extent (Hopkins et al., 2015).

Given PARP1’s involvement in single strand break repair, it was proposed that PARP1 trapping results in a DNA lesion that cannot be bypassed by replication forks (Farmer et al., 2005). Subsequently, leading to the formation of DSBs and stalled replication forks at the site of damage, as the cell progresses through S-phase (Solier and Pommier, 2014). DSBs can only be repaired through homologous recombination (HR) repair or non-homologous end joining (NHEJ). As previously discussed, HR is essential for the error-free repair of DSBs and requires functional BRCA1/2 proteins (Offit, 2006; Palomba et al., 2014; Vos et al., 2018; Bu et al., 2019). In HR deficient tumors, such as BRCA1/2 mutated tumors, the inhibition of PARP yields DSBs which can only be repaired through NHEJ. NHEJ mediates the direct re-ligation of DNA lesions without the requirement of a homologous template. This direct re-joining increases the incidence of catastrophic genomic instability which may result in cell death. Furthermore, PARPi-induced collapsed replication forks cannot be repaired by NHEJ, resulting in death in HR-deficient tumor cells (Figure 1b) (Min A. et al., 2013).

Several studies have examined the correlation between PARP-trapping and tumor sensitivity. The main evidence supporting this mechanism is that the PARP-trapping activity of PARP inhibitors correlates with their cell line toxicity (from the most to the least potent): Talazoparib > > Niraparib > Olaparib = Rucaparib > > Veliparib (Murai et al., 2012a; Murai et al., 2014a). This mirrors the cytotoxicity observed in tumor cell lines, with Talazoparib being active at nanomolar concentrations and Veliparib remaining inactive at 100 μM.

A recent study used a modified proximity ligation assay to detect chromatin-trapped PARP1 and concluded that PARP1 trapping correlated with cellular toxicity in both non-malignant and tumor cells, which may limit the therapeutic advantage of potent trapping activity. It was also observed that three different PARP inhibitors caused similar tumor growth inhibition, regardless of their PARP-trapping potency, suggesting that PARP-trapping may not entirely mediate the anti-cancer activity of PARP inhibitors (Hopkins et al., 2019). Consistent with the conclusions from this study, the link between PARP-trapping and tumor toxicity remains unclear in clinical studies. Veliparib, which was determined to have the lowest PARP-trapping activity, was shown to have a response rate of 47% in patients with platinum-resistant or partially platinum-sensitive BRCA-mutated epithelial ovarian cancer (Vergote et al., 2015). This was comparable to the response rate of platinum sensitive/resistant or BRCA-mutated ovarian tumors to Niraparib (40%) (Sandhu et al., 2013), Olaparib (46%) (Fong et al., 2010), and Talazoparib (42%) (de Bono et al., 2017).

Therefore, although it is tempting to speculate that PARP-trapping mediates its anti-cancer activity, there is a lack of clinical evidence to support this theory. Specifically, the extent of each PARP inhibitor’s PARP:DNA trapping capacity does not correlate clearly with the overall toxicity of each drug in the clinic, suggesting that other factors are also involved.

### Activation of the Non-homologous End Joining Repair Pathway

Several studies have suggested that the synthetically lethal interaction between BRCA1 and PARP inhibition is due to the upregulation of NHEJ activity in HR-deficient tumor cells. This hyper-activation of NHEJ increases the likelihood of catastrophic genomic instability and subsequent cell death (George et al., 2017). This was initially hypothesized following the finding that PARPi treatment increases the phosphorylation of DNA-PK substrates, consequently promoting NHEJ activity (Figure 1c) (Patel et al., 2011). In support of this theory, studies have shown that anionic poly (ADP-ribose) (pADPr) scaffolds produced by PARP1 activation directly interact with Ku70 and Ku80 to inhibit classical NHEJ (Scott et al., 2015). Thereby, inhibiting PARP1’s activity removes this negative regulation to promote the upregulation of NHEJ activity. Furthermore, Veliparib treatment was also shown to enhance NHEJ activity in BRCA-deficient ovarian carcinoma cell lines (Patel et al., 2011). This was further supported by another study which demonstrated that depletion of several NHEJ proteins, including DNA-PK and Ku80, induced PARPi resistance in previously sensitive cell-based models (Choi et al., 2016).

Shieldin has been recently identified as a 53BP1 effector complex that is recruited to DSBs via the ATM-RNF8-RNF16-53BP1-RIF1 axis (Dev et al., 2018). Shieldin recruitment at the site of damage has been shown to promote NHEJ activity, fusion of unfinished telomeres and class-switch recombination (CSR) (Greenberg, 2018). Deletion or inhibition of Shieldin, 53BP1, RIF1 or REV7 has been shown to correlate with increased PARPi resistance (Xu et al., 2015; Francica and Rottenberg, 2018; Gupta et al., 2018). Furthermore, a recent study demonstrated TRIP13 ATPase acts as a negative regulator of REV7 via catalyzing the conformational transformation of REV7 to an inactive state. It was also observed that tumors with elevated expression of TRIP13 ATPase possessed significant Olaparib resistance, mediated by the down regulation of REV7 activity (Clairmont et al., 2020). Given the finding that Shieldin activity directly promotes NHEJ, this correlation supports the hypothesis that PARPi lethality is due to the hyper-activation of NHEJ activity.

In contrast, simultaneous treatment with a DNA-dependent protein kinase (DNA PKcs) inhibitor (AZD7648) and a PARPi (Olaparib) has been shown to have synergistic effects in BRCA mutated tumor cells (Fok et al., 2019). It was hypothesized that this was due to the catastrophic genomic instability induced by concurrent inhibition of NHEJ via the DNA PK inhibitor and the pre-existing HR defect of these cells. This finding suggests that the PARPi mechanism is not fully described by the NHEJ activation theory, given that suppression of NHEJ would be predicted to induce PARPi resistance in these circumstances.

### Disrupted Processing of Okazaki Fragments and Replication Fork Speed

It was recently demonstrated that inhibition or depletion of the replication fork regulators, FEN1 and LIG1, results in PARP1 accumulation, thereby enabling XRCC1-mediated processing (Hanzlikova et al., 2018). Supporting a role for PARP1 in responding to unligated Okazaki fragments, it has also been found that PARPi therapy increased replication fork progression speed by 1.4-fold (Figures 1d,e) (Maya-Mendoza et al., 2018). This suggests an underlying mechanism of PARPi toxicity could be the result of DSBs occurring as a result of high-speed replication (Maya-Mendoza et al., 2018; Quinet and Vindigni, 2018). Based on these findings, it was also recently proposed that increased replication speed may result in the accumulation of replication-associated single-stranded DNA (ssDNA) gaps (Cong et al., 2019). It was hypothesized that these cytotoxic ssDNA gaps were attributed to PARP1’s role in processing Okazaki fragments or the reversal of stalled replication forks. Therefore, inhibiting the action of PARP within these processes would result in the formation of short single-stranded gaps in the DNA sequence. Although not yet well recognized, this theory does possess significant supporting evidence. This includes the substantially increased prevalence of ssDNA gaps following PARPi treatment in BRCA-deficient tumor cell lines, in comparison to those that were BRCA-wild type. Furthermore, significantly less ssDNA gaps were observed in PARPi resistant cell models, demonstrating that PARPi sensitivity correlates with the level of ssDNA gaps induced by PARPi treatment (Cong et al., 2019).

### Disruption of the Role of PARP1 in Transcription

In addition to roles in DNA repair, PARP1 also regulates the transcription of several proteins, by mechanisms such as regulating chromatin structure and histone PARylation, directly acting as transcriptional co-regulator and direct binding to transcription sites (Schiewer and Knudsen, 2014). As such, PARP1 also regulates the transcription of several proteins implicated in cancer cell survival, including p53 and NF-κB (Stanisavljevic et al., 2011; Lee et al., 2012). Therefore, inhibition of PARP1 using PARPi could also lead to the inhibition of oncogenes regulated by PARP-dependent transcription. An example of this is the sensitization of Ewing’s sarcoma by PARPi, in part due to the inhibition of PARP-dependent transcription of ETS gene fusions such as EWS-FLI-1 (Brenner et al., 2012). PARPi treatment also reduces the transcription of DDX21, which leads to the inhibition of rDNA transcription and ribosome biogenesis in BRCA1/2 proficient breast cancers leading to reduced cancer growth (Kim et al., 2019).

In conclusion, multiple mechanisms have been proposed to mediate PARPi toxicity in BRCA1/2 mutated tumors since their initial discovery and clinical application. However, it is not yet established whether one or several of these mechanisms mediate the anti-tumor effects induced by PARPi therapy and further study is required to increase our understanding. It is considered likely that PARPi-induced inhibition of the repair of DNA single-strand breaks and PARP-trapping contributes to the collapse of replications forks, but that other mechanisms are also likely to be involved.

## PARPi Resistance

A major complication associated with anti-cancer therapies is the development of acquired resistance in tumors. Human and rodent models have shown that the extent of initial responsiveness to PARPi therapy correlates with the severity of resistance. Therefore, this suggests that individuals who are more likely to see a substantial effect during initial PARPi treatment are most likely to experience poor long-term sensitivity.

### Restoration of HR Activity

One of the most well-established mechanisms of acquired PARPi resistance is through the restoration of HR capacity. Through restoring HR capacity, DSBs can be effectively repaired, and the tumor cell continues to survive. This mainly occurs as a result of reversion mutations or the suppression of NHEJ activity.

#### Reversion Mutations

The most frequent method by which HR is restored is by the reactivation of BRCA1/2 due to secondary mutations. These reversion mutations have been identified in patients diagnosed with both germline and somatic BRCA1/2 mutated breast and ovarian carcinomas (Shroff et al., 2018). A study of high-grade ovarian cancers showed BRCA reversion mutations were identified in the circulating cell-free DNA of 18 and 13% of platinum-refractory and platinum-resistant tumors, respectively. Furthermore, the presence of a BRCA1/2 reversion mutation was shown to have decreased the PFS induced by Rucaparib treatment from 9 to 1.8 months (Lin et al., 2019). This provided the first clinical evidence that intragenic deletions of BRCA1/2 contribute to the development of PARPI resistant tumors.

Open reading frame (ORF) mutations result in BRCA function being restored due to the removal of the initial delirious mutation and subsequently, result in HR being reactivated (Christie et al., 2017). These reversion mutations have been observed in both patient samples and cellular based studies. For instance, a 55-year-old woman was diagnosed with an ER+ metastatic breast cancer that initially showed sensitivity to Olaparib treatment due to a V1283fs^∗^2 mutation in BRCA2, which is a recognized loss of function mutation. However, after approximately 10 months of treatment the patient’s primary tumor showed Olaparib resistance. A circulating tumor DNA assay was conducted on the patient’s blood sample and a secondary BRCA2 D1280_N1288 deletion mutation was detected. This mutation is predicted to restore the ORF function via the deletion of the V1283fs^∗^2 BRCA2 mutation, without the removal of critical components of the gene (Gornstein et al., 2018). Therefore, creating a functional isotype of BRCA2 which induces PARPi resistance in previously sensitive cellular models by restoring effective HR (Edwards et al., 2008). Similarly, the c.6174d deletion mutation is a BRCA2 mutation frequently observed in the Ashkenazi Jewish population, which results in truncated BRCA2 protein and confers PARPi sensitivity (Wang and Figg, 2008). Several intragenic mutations which cause the deletion of the c.6174d mutation and subsequently restore the ORF function have been identified in cellular models (Edwards et al., 2008).

However, further genetic testing of BRCA status following acquired PARPi resistance is infrequent, resulting in the cause of resistance commonly remaining undiagnosed (Jiang et al., 2019). This is often disadvantageous to the patient as knowledge of these mutations may guide treatment opportunities. For instance, treatment with the chemotherapeutic agent, 6-Thioguanine, has been shown to be effective at overcoming PARPi resistance induced by BRCA2 reversion mutations (Issaeva et al., 2010). Similar reversion mutations have been observed in patients who were previously sensitive to PARPi therapy due to mutations in RAD51C or RAD51D (Kondrashova et al., 2017).

#### Suppression of Non-homologous End Joining

Several papers have shown that defective HR resulting from BRCA1 mutations can be reactivated due to concomitant disruption of genes which regulate NHEJ (Noordermeer and van Attikum, 2019). Depletion of 53BP1, a protein involved in the activation of NHEJ, rescues BRCA1-deficient HR and decreases hypersensitivity to PARP inhibitors (Bouwman et al., 2010). Furthermore, as discussed above, the Shieldin complex has been identified as a 53BP1 effector complex. Reduced expression of Shieldin has been observed in numerous breast carcinomas exhibiting acquired PARPi resistance. In addition, REV7 localize to the site of damage following a DSB and is known to promote NHEJ activity and suppress HR (Xu et al., 2015). Inhibition of REV7 via shRNA has been shown to inhibit NHEJ and consequently, promote HR. This shRNA mediated inhibition of REV7 induces PARPi resistance and rescue cells from Olaparib-induced cytotoxicity (Clements et al., 2019). In support of this theory, elevated expression of TRIP13 ATPase has been identified in a large cohort of PARPi resistant BRCA1 mutated carcinomas. As previously discussed, TRIP13 ATPase indirectly suppresses NHEJ activity via the down regulation of REV7. Increased Olaparib sensitivity was also observed in TRIP13 depleted cellular models; therefore, further supporting the hypothesis that TRIP13 ATPase is involved in mediating sensitivity to PARP inhibitors via regulating NHEJ activity (Clairmont et al., 2020).

microRNAs are small, highly conserved regions of non-coding RNA, recognized to have a role in regulating gene expression (Macfarlane and Murphy, 2010). A recent screen revealed that increased expression of miR6-22, miR644, miR-492, miR-613, miR-577, and miR-126 were associated with PARPi resistance (Choi et al., 2014, 2016). However, only over-expression of miR-622 was shown to desensitize BRCA-mutated breast and ovarian cancer cell lines to Olaparib and Veliparib treatment. It was proposed that this desensitization is due to the miR-622 mediated down regulation of Ku 70/80 expression; thereby, blocking NHEJ activity and promoting HR activity (Choi et al., 2016). Collectively, the above findings support the hypothesis that down regulation of NHEJ may play a role in PARPi resistance due to upregulation of HR activity.

### Increased Drug Efflux

Increased drug efflux is where there is an increase in the rate which compounds, such as PARP inhibitors, are removed from cells. There is some evidence which suggests that PARPi resistance may be due to increased expression of drug efflux transporter genes. It is hypothesized that this is specifically mediated by the ATP Binding Cassette Subfamily B Member 1 and 2 (Abc1a/b) genes, with one study showing that expression of Abcb1a/b was increased by 2- to 85- fold in Olaparib resistant breast cancers (Rottenberg et al., 2008). Furthermore, Abc1a/b expression was shown to be correlated with resistance to Olaparib and Rucaparib treatment in ovarian cancer cell lines. This resistance was reversed following treatment with Verapamil or Elacridar, two commonly prescribed Abcb1a/b inhibitors (Vaidyanathan et al., 2016). However, Abcb1a/b over-expression was not shown to induce resistance to treatment with Veliparib or AZD2461, an Olaparib analog, AZD2461 indicating that this is unlikely to be the sole mechanism of PARPi resistance (Vaidyanathan et al., 2016).

### Stabilization of Stalled Replication Forks

The stabilization of stalled replication forks inhibits their collapse and the subsequent creation of double stranded breaks (Taglialatela et al., 2017). Pre-clinical evidence has indicated that this stabilization may contribute to the acquired PARPi resistance experienced by patients. This was initially proposed by Chaudhuri et al. (2016), following the discovery that depletion of the MLL3/4 complex protein, PTIP, prevents PARPi induced replication fork stalling in BRCA-deficient cells. Following its localization at the site of replication, PTIP recruits MRE11 to the site of damage to promote the degradation of stalled replication forks. Consequently, restarting the stalled replication fork and improving resection at the site (Ying et al., 2012; Chaudhuri et al., 2016). Therefore, depletion of PTIP inhibits the recruitment of MRE11 to the stalled replication fork to minimize degradation of the nascent strand of DNA. This results in less replication fork collapse associated DSBs in BRCA1/2 deficient cells and confers PARPi resistance.

EZH2 is a histone methyltransferase and catalytic subunit of PRC2, proposed to contribute to PARPi efficiency (Yamaguchi et al., 2018). PARP1 is known to activate and PARylate EZH2, causing it to dissociate from PRC2 and later, degrade. Following replication fork stalling, EZH2 localizes to the fork and promotes the methylation of histone H3. This methylation facilitates the recruitment of a nuclease, MUS81, to the replication fork to promote replication fork degradation (Rondinelli et al., 2017). Depletion, or deactivation, of EZH2 or MUS81 has been shown to induce PARPi resistance by promoting replication fork stabilization.

### Down-Regulation of PARG Protein Expression

As previously discussed, PARP1 undergoes auto-PARylation promote its full activation and promote the PARylation of other chromatin-associated proteins. PARylation has been well characterized as a reversible post-translational modification, with Poly (ADP-ribose) glycohydrolase (PARG) identified as the primary PAR degrading enzyme (Miwa and Sugimura, 1971). PARG functions via hydrolyzing the ribose-ribose bond to produce adenosine diphosphate (ADP) ribose (Miwa et al., 1974).

*In vitro* and *vivo* findings have demonstrated PARG depletion is a common occurrence in PARPi resistant BRCA2-deficent mouse mammary tumor models (Gogola et al., 2018). Given PARPi have been proposed to significantly inhibit PARylation, it is hypothesized that depletion or inactivation of PARG enables PAR accumulation to maintain adequate PARP function, preventing PARP trapping and promoting PARPi resistance. However, further study is required to determine whether changes in PARG levels is a mechanism of PARPi resistance in human cancers.

Notably, several PARG inhibitors (PARGi) are currently undergoing pre-clinical development. Several studies have shown promising anti-tumor outcomes when utilizing combination PARPi/PARGi treatment in PARPi-resistant glioblastoma and cellular models (Houl et al., 2019).

## Combination Treatments

Given high dosage requirements and the prevalence of acquired PARPi resistance, combination therapies are of significant interest to minimize dosage requirements and increase drug efficiency.

### PARP Inhibitors and Alkylating Agents

Cytotoxic chemotherapy using alkylating agents remains one of the most frequently utilized anti-cancer therapies. Alkylating agents are a class of chemotherapeutic drugs which induce cell death by directly adding additional alkyl groups to the bases of DNA, most frequently via the N7 position on guanine residues (Damia and D’Incalci, 1998). This results in significant intra- and inter- strand linking at the alkylated residues to induce DNA damage. In cancer cells, undergoing rapid growth, this leads to inhibition of DNA replication, cell division and subsequent cell death. Alkylating agents frequently utilized in cancer therapy include the platinum compounds Cisplatin and Carboplatin, and Temozolomide. Platinum compounds crosslink the purine bases within DNA, inducing DNA damage.

Although these drugs initially show beneficial anti-cancer activity, most tumors develop acquired or *de novo* mutations resulting in chemo-resistance and poor patient outcomes. Furthermore, many patients require high dosages for effective tumor size reduction following the administration of chemotherapy alone. This results in a large proportion of patients experiencing adverse side effects, which decreases their quality of life during treatment. Therefore, there is a clear requirement for combination therapies in order to decrease the dosage of chemotherapy. PARP inhibitors have been demonstrated to be novel chemotherapeutics and chemopotentiators.

Early studies of PARP inhibitors with platinum chemotherapy showed higher levels of myelosuppression and it was suggested that this could be linked to the trapping ability of PARP inhibitors. Therefore, it was proposed that, due to its lower PARP trapping activity, Veliparib may be less myelotoxic than other PARP inhibitors. The Phase III VELIA trial recently showed that Veliparib in combination with chemotherapy for first-line and maintenance treatment of stage III or IV high-grade serous ovarian cancer significantly improved progression-free survival (PFS) (Coleman et al., 2019). Furthermore, the phase III BROCADE3 trial showed that 34% of HER2-negative, BRCA-mutated breast cancer patients treated with Veliparib, Carboplatin, and Paclitaxel were progression free at 24 months, compared to 20% of patients treated with Carboplatin and Paclitaxel alone (Han et al., 2017). To further support this, the Phase III PAOLA trial showed Veliparib in combination with Carboplatin or Paclitaxel in HER2-negative advanced or metastatic germline BRCA-mutated breast cancer significantly improved PFS without notably increasing toxicity (Ray-Coquard et al., 2019). Additionally, the Phase III PRIMA study of recurrent platinum sensitive BRCA-mutated ovarian cancer patients showed that Niraparib significantly improved median progression free survival following platinum-based chemotherapy, in comparison to patients treated with a placebo. Patients with BRCA wild-type tumors showed a PFS of 13.8 months following Niraparib maintenance therapy, in comparison to 8.2 months for those administered a placebo (Gonzalez-Martin et al., 2019). This demonstrates the effectiveness of maintenance PARPi treatment following chemotherapy in ovarian tumor, regardless of BRCA status (Gonzalez-Martin et al., 2019).

The alkylating agent Temozolomide acts by adding methyl groups to guanine at the O6 and N7, and adenines at the N3 positions, leading to single-strand breaks (SSBs) at the N7 methylated guanines and N3 methylated adenines (Zhang et al., 2012). These Temozolomide-induced SSBs require PARP1 for repair and therefore induce PARP1-recruitement, which is subsequently trapped in the presence of PARP inhibitors (Murai et al., 2014b). In light of this increased PARP1 trapping in the presence of Temozolomide, it is proposed that the synergy observed between the two treatments is dependent upon inhibition of PARP’s catalytic activity and trapping potential of PARP inhibitors. This is supported by preclinical studies which show that Talazoparib and Olaparib have a greater synergistic affect with Temozolomide than Veliparib or genetic inactivation of PARP1/2 (Murai et al., 2014b). As previously discussed, increased PARP trapping has been proposed to contribute toward myelosuppression and in support of this, a phase II clinical trial combining Rucaparib and Temozolomide, observed increased myelosuppression patients with metastatic melanoma (Plummer et al., 2013). It has since been suggested that this combination treatment will require a truncated PARPi treatment schedule, to minimize the negative effects on bone marrow function.

### PARP Inhibitors and Topoisomerase I Inhibitors

Topoisomerase I (TOP1) is an enzyme that functions to reduce torsional stain on the DNA helix by the induction of single-strand breaks. Inhibition of topoisomerase I by the Camptothecin related compounds, Topotecan or Irinotecan, traps TOP1 on the DNA leading to single-strand breaks that are then converted into double-strand breaks during the S-phase of the cell cycle resulting in tumor cell death (Xu and Her, 2015). In contrast to alkylating agents, the synergistic effects of topoisomerase inhibitors and PARP inhibitors do not depend on the PARP-trapping activity. Instead the synergy is suggested to result from 3 main mechanisms, firstly, the inhibition of TOP1-PARylation, which is required for the release of trapped TOP1 (Malanga and Althaus, 2004). Secondly, the inhibition of HR and stimulation of NHEJ (Maede et al., 2014) and thirdly, the inhibition of tyrosyl-DNA-phosphodiesterase 1 (TDP1), which is the enzyme required for the cleavage of TOP1-covalently linked complexes from the DNA (Das et al., 2014). It remains to be determined whether this combination confers a therapeutic advantage in the clinic compared to either inhibitor alone.

### PARP Inhibitors and WEE1 Kinase Inhibitors

WEE1 kinase is a critical cell cycle regulator protein, involved in G2-M cell cycle arrest prior to mitotic entry. Therefore, inhibition of WEE1 promotes the rapid progression through the cell cycle to inevitably produce genomic instability which subsequently results in mitotic catastrophe and cell death (Matheson et al., 2016). Initial investigations of WEE1i/PARPi simultaneous combination treatments showed disappointing outcomes, due to overwhelming toxicity to non-malignant cells being poorly tolerated in mouse studies. However, sequential WEE1i/PARPi treatment was shown to have significant additive anti-tumor effects in xenograft models, whilst minimizing replication stress induced in non-malignant tissue; therefore, decreasing off-target toxicity (Fang et al., 2019). Furthermore, low dose WEE1i and PARPi combination treatment has shown to act as a radiosensitizer in pancreatic cancer and KRAS-mutated NSCLC models (Karnak et al., 2014; Parsels et al., 2018).

### PARP Inhibitors and PI3k Inhibitors

Phosphoinositide 3-kinases (PI3ks) are a class of enzyme involved in numerous cellular processes, including proliferation, intracellular trafficking and differentiation. The use of PI3k inhibitors in cancer therapy has been well established, given the PI3k pathway has been suggested to be one of the most commonly activated pathways in cancer cells (Liu et al., 2009). In cellular Ovarian cancer models, combination treatment with a PI3ki, Buparlsib, and Olaparib has been shown to significantly inhibit cellular proliferation by downregulating BRCA1/2 expression. This effect was observed in BRCA wild-type cell lines which did not possess PIK3CA mutations, providing a rationale for the use of this combination in a wider cohort of tumors independent of their mutational status (Wang et al., 2016). Furthermore, cellular and xenograft models have shown promising results for the use of PARPi and PI3ki combination therapy in the treatment of PTEN/p53-deficient prostate cancer models (González-Billalabeitia et al., 2014). Similar down-regulation of BRCA1/2 and subsequent PARPi sensitivity has also been observed in BRCA-wildtype TNBC cellular studies following treatment with a Buparlsib and Olaparib combination (Ibrahim et al., 2012).

### PARP Inhibitors and Radiation

PARP inhibitors have been shown to radio-sensitize tumor cells in several studies, irrespective of BRCA status (Zhao W. et al., 2019). It is proposed that the underlying mechanism for this sensitization is that PARP inhibitors inhibit the repair of radiation-induced single-strand breaks, leading to replication fork collapse and subsequent DSBs in S-phase (Dungey et al., 2008).

Several clinical trials have been conducted to establish the efficacy of radiation therapy in combination with PARPi treatment; however, clinical data from these studies have not yet been published. A phase II trial in patients with brain metastases from non-small cell lung cancer, combining whole brain radiotherapy with Veliparib, observed no clinical benefit over whole brain radiotherapy plus a placebo (Chabot et al., 2017).

### PARP Inhibitors and Immunotherapy

Immunotherapies are an emerging class of cancer therapy, showing promising results as both monotherapies and combination therapies. During the initiation of the innate immune response, pattern recognition receptors (PRRs) recognize pathogen-associated molecular patterns (PAMPs), and damage-associated molecular patterns (DAMPs) (Amarante-Mendes et al., 2018). PAMPs are small molecule motifs conserved within a class of microbes; therefore, are not stimulated by PARPi treatment. However, DAMPs are endogenous molecules released from host cells during damaging or death related cellular events (Huang et al., 2015). Cytosolic DNA, which arises due to nuclear damage or loss-of-function mutations in DNA degrading proteins, has been identified as a DAMP which can bind to cyclic guanosine monophosphate (GMP)– adenosine monophosphate (AMP) synthase (cGAS) to induce a conformational change in cGAS (Li and Chen, 2018). This conformational change results in the conversion of guanosine triphosphate (GTP) and ATP to the second messenger, cyclic GMP-AMP. GMP-AMP is then able to act as an endogenous ligand for Stimulator of IFN Gene (STING), which activates numerous transcription factors to stimulate an innate immune response (Kato et al., 2017).

Based on evidence of an interaction between the DNA damage response and the immune system, it has been suggested that PARPi therapy may have positive implications for the anti-cancer immune response (Li and Chen, 2018). It is now well recognized that tumors with mutations in DNA damage response genes are more sensitive to immunotherapies (Samstein and Riaz, 2018). For instance, a study of patients diagnosed with advanced urothelial carcinomas demonstrated that the presence of mutations in DNA damage response genes increased the response to PD-1/PD-L1 blockade therapies by 4.2-fold (Vidotto et al., 2019). To further expand on the above findings, preclinical studies showed that Talazoparib and Veliparib treatment induced catastrophic DNA damage which activated cGAS (Chabanon et al., 2019; Pantelidou et al., 2019).

Interestingly, a recent study demonstrated that PARPi treatment induced STING activation in cellular models deficient of BRCA2 via shRNA technology; however, this was not observed in BRCA-proficient cells (Reisländer et al., 2019). These findings were controversial; given they suggest that immune checkpoint inhibitors were unlikely to be effective in combination with PARP inhibitors in HR-proficient individuals.

Several clinical trials are currently underway investigating the effects of PARP inhibitors in combination with PD-1 inhibitors. Results from a Phase I study were 49 patient’s suffering from solid tumors were treated with a combination of a PARPi and Tislelizumab showed 20% of patients achieved an objective response. Furthermore, 32% of patients entered a state of stable disease, where the tumor did not show any increase in size (Friedlander et al., 2019).

### PARP Inhibitors and Drugs Targeting Epigenetic Modifications: DNA Methyltransferase Inhibitors (DNMTi)

DNA methyltransferases (DNMTs) are a conserved family of enzymes, responsible for the transfer of methyl groups via *S*-adenosyl methionine (SAM). DNMTs have a vital role in gene silencing, transcriptional activation and post-transcriptional gene regulation (Lyko, 2018). Deregulated DNMT function has been associated with numerous components of cancer development, including silencing of tumor suppressor genes and hypermethylation of cancer-associated genes. For instance, hypermethylation of the retinoblastoma gene promoter region has been observed in a significant number of unilateral retinoblastoma cancers (Robertson, 2001). Dysregulation of DNMT activity, and subsequent hypermethylation of promoter regions, has been identified as a key component in acute myeloid leukemia initiation and progression (Yang et al., 2019). Furthermore, hypermethylation of promoter regions has also been observed in 56% of breast and 15–30% ovarian cancers (de Almeida et al., 2019; Hentze et al., 2019).

Given the clear link between excessive DMNT activity and tumorigenesis, the development of DNMT inhibitors offered a promising, targeted anti-cancer therapy via inhibiting the methylation of DNA residues. Currently, two DNMT inhibitors, Azacytidine (Vidazaand) and Decitabine (Dacogen), that have received FDA and European Medicines Agency approval for the treatment of acute myeloid leukemia and myelodysplasia syndrome. However, impartial or no response is experienced by greater than 50% of patients undergoing DNMT inhibitor therapy. This indicates the need for a more targeted, potent approach to DNMT inhibitor therapy. Reversing the gene expression changes associated with DNA methylation abnormalities in cancer is one proposed mechanism for the clinical efficacy of DNMTis (Baylin and Jones, 2011). It has also been determined that DNMTi can be incorporated into replicating DNA in place of cytosine bases. Once added to DNA, these can then covalently bind DNMTs, effectively trapping DNMT on the DNA leading to cell death (Chovanec et al., 2018). It has been observed that PARP can bind to DNMT and therefore treatment with both PARPi and DNMTi increase PARP trapping on the DNA. DMNT inhibitors have also been shown to increase the accumulation of reactive oxygen species (ROS). This increase in oxidative stress activates cellular kinase activity to promote PARP1 binding at the site of damage. Therefore, promoting the trapping of PARP1 at site of damage via PARP inhibitors and the subsequent replication fork collapse (Pulliam et al., 2018).

Recent pre-clinical cellular and xenograft breast cancer and AML studies using a PARPi and DNMT inhibitor (DNMTi) combination have shown promising outcomes, including decreased clonogenic formation and increased cytotoxicity (Muvarak et al., 2016). Furthermore, a recent study demonstrated that combination Guadecitabine and Talazoparib therapy enhanced PARPi trapping activity in cellular assays, and decreased tumor growth in ovarian and TNBC xenograph models (Pulliam et al., 2018). The PARPi:DNMTi combination therapy has not yet been trialed in the clinic but a phase I/II trial is currently recruiting patients to assess the efficacy of Talazoparib in combination with the DNMTi, Decitabine, for treatment of acute myeloid leukemia.

## Clinical Significance: PARP Inhibitors as a Cancer Therapy

PARP inhibitors have shown promising results in both clinical trials and practice for the treatment of ovarian, breast, prostate and pancreatic cancers. There are currently 286 clinical trials registered on clinicaltrials.gov investigating PARPi therapies.

### Ovarian Cancer

As discussed previously, BRCA1/2 mutations have been identified in approximately 10–15% of ovarian cancers (Bryant et al., 2005). The benefit of PARP inhibitors as a maintenance therapy for ovarian cancer has been well established, since the approval of Olaparib in 2014 (reviewed in Franzese et al., 2019). However, recent studies have shown that PARPi can also have clinical benefit as a first line therapy in ovarian cancer treatment.

In the recent PRIMA phase III randomized trial, 733 patients with newly diagnosed ovarian cancer were treated with Niraparib or placebo, following a response to platinum-based chemotherapy. The study outcomes showed that median PFS was significantly longer in the niraparib-treated group than in the placebo group (21.9 months vs. 10.4 months). Significantly, this increase in PFS was higher in HR deficient tumors but an increase in PFS was still observed in HR proficient tumors (Gonzalez-Martin et al., 2019).

The recent VELIA Trial aimed to assess Veliparib as a font line therapy for Ovarian cancer. Over 1000 women with newly diagnosed ovarian cancer were assigned first line therapy of chemotherapy plus either Veliparib or placebo followed by maintenance therapy of Veliparib or placebo. Veliparib was found to extend median progression free survival by 7 months over all (24 months vs. 17 months). The PFS was improved further in patients with BRCA mutations (35 months vs. 22 months), suggesting that PARPi could be an efficient front-line therapy for ovarian cancer (Coleman et al., 2019).

### Breast Cancer

Approximately 5–10% of breast cancer cases are due to inherited genomic alterations. Similar to ovarian cancer, the majority are caused by BRCA1/2 mutations (Lee et al., 2020). For individuals with a BRCA1/2 mutation, the risk of developing breast cancer is 69 and 62%, respectively. However, the risk for individuals without a BRCA mutation is as low as 12% (Armstrong et al., 2019). The phase III OlympiAD trial demonstrated that maintenance therapy with Olaparib significantly increased PFS in patients with metastatic HER2-negative BRCA-mutated breast cancer, in comparison to standard chemotherapy (Robson et al., 2017, 2019). Given these findings, Olaparib was approved by the FDA in 2018 for the treatment of metastatic HER2-negative BRCA-mutated breast cancer following chemotherapy (Le and Gelmon, 2018). In 2018, the TALA study provided the first evidence that Talazoparib could induce a complete pathological response as a monotherapy treatment in the treatment of BRCA-mutated breast cancer. This was further supported by the phase III EMBRACA study which demonstrated that Talazoparib monotherapy had significantly greater PFS in patients with metastatic HER2-negative BRCA-mutated breast cancer in comparison to standard chemotherapy treatment (Litton et al., 2018). Given this, the FDA approved Talazoparib as the second PARPi for the treatment of breast cancer (Litton et al., 2018). Additionally, patient-reported studies have shown PARPi therapy offered significantly greater patient quality of life during treatment in comparison to several standard therapies (Ettl et al., 2018; Hurvitz et al., 2018). Collectively, these findings highlight the potential of PARP inhibitors as viable breast cancer treatment.

### Prostate Cancer

Prostate cancer accounts for 7.1% of all cancer diagnoses in men, although contributes to an unproportionable 13.3% of cancer related deaths (Crawford, 2003). Improvements have been made for treatment options, although a radical prostatectomy remains the gold standard treatment. Radical prostatectomies are minimally invasive procedures, although many patients experience long-term side effects that significantly decrease their quality of life (Chin, 2009). Therefore, there is a clear requirement for alternative treatment options to be made available. The application of PARP inhibitors in the treatment of prostate cancer was initiated in 2015 following the finding that 19.6% of prostate cancers had BRCA1, BRCA2 or ATM mutations (Mandelker et al., 2017). Currently, numerous clinical trials are being completed to investigate the effectiveness of PARPi mono- and combination therapies in the treatment of prostate cancer. The phase II TOPARP study showed that following treatment with 400 mg Olaparib, 54.3% of patients with DNA repair mutated, castration-resistant prostate cancer had a composite response at a two-year follow up (Mateo et al., 2020). The Phase II Galahad study investigated the effect of Niraparib treatment in patients suffering from metastatic castration-resistant prostate cancer which possessed a DDR defect. The results demonstrated that 65% of patients diagnoses with a BRCA1/2-mutated prostate carcinoma, and 31% of patients with alternative DDR gene mutated prostate cancers, achieved a composite response (Smith et al., 2019).

### Pancreatic Cancer

Pancreatic cancer is recognized to be one of the most common cause of cancer-associated deaths worldwide, with the 5-year survival rate being a mere 9% (Rawla et al., 2019). Due to its asymptomatic progression, most patients do not present until advanced-stage disease. Although surgical and adjuvant pancreatic cancer treatments are advancing, the 5-year survival statistics continue to worsen (Brunner et al., 2019). This highlights the urgent need for the development of effective, targeted anti-cancer therapies to improve patient survival (Brunner et al., 2019). BRCA1/2 mutations have been identified in 4–7% of pancreatic cancer patients. Furthermore, these mutations have been correlated with poorer survival outcomes in pancreatic cancer patients (Iqbal et al., 2012). The recent POLO trial showed that in patients with chemotherapy responsive BRCA1/2-mutated tumors, 22.1% of patients treated with Olaparib did not have any tumor progression after two years. In contrast, only 9.6% of patients treated with the placebo showed no tumor progression. Furthermore, the median PFS was determined to be 7.4 and 3.8 months following Olaparib and control drug treatments, respectively (Golan et al., 2019). This clinical trial provided the first evidence for the effectiveness of PARP inhibitors in the treatment of pancreatic cancer and subsequently resulted in the FDA approval of Olaparib for the treatment of germline BRCA1/2-mutated metastatic pancreatic adenocarcinomas.

### Lung Cancer

Lung cancer accounts for 2.09 million of annual cancer diagnoses and is the leading cause of worldwide cancer-associated deaths (Cao and Chen, 2019). DDR mutations are evident in a significant proportion of lung cancer patients, including mutations in ATM, PTEN, MRE11, and FANCA (Mamdani et al., 2019). Most notably, 5% of lung cancers have been identified to be BRCA1/2-mutated. Collectively, these findings provide a rationale for the use of PARP inhibitors in the treatment of lung cancer. However, the phase II STOMP trial demonstrated that maintenance Olaparib monotherapy for small cell lung cancer (SCLC) did not significantly increase PFS or overall survival, in comparison to a placebo. Subsequently, the phase I/II clinical trial examining the effectiveness of an Olaparib/Temozolomide combination treatment in reoccurring SCLC demonstrated that 41.7% of participants had a complete pathological response (Farago et al., 2019).

PARP inhibitors are well recognized to induce radio-sensitization in various cancer subtypes. However, cellular and xenograft-based studies provided the first evidence that Talazoparib sensitizes a significant proportion of NSCLC models to ionizing radiation. A similar effect was also observed following Veliparib treatment; however, to a lesser extent. Given Talazoparib has a significantly greater PARP trapping capacity, it is hypothesized that PARP trapping may be the underlying mechanism by which sensitivity to radiation is induced (Laird et al., 2018). Fluzoparib has been identified as a novel PARPi, in the early stages of preliminary clinical trials (Wang et al., 2019). Fluzoparib has shown promising results in Phase I/II lung cancer clinical trials as a radiosensitizer and in combination with SHR-1316, a PD-L1 inhibitor (Luo et al., 2019).

### Acute Myeloid Leukemia

Acute myeloid leukemia (AML) is the most common cause of adult leukemia, contributing to 80% of adult leukemia diagnoses (Yamamoto and Goodman, 2008). Although BRCA1/2 mutations are not characteristic of AML, several pre-clinical studies have demonstrated genomic mutations which provide a rationale for PARPi use in AML therapy (reviewed in Faraoni et al., 2019). It was initially shown that microsatellite instability-positive AML cellular models exhibited down-regulation and mutation of the HR genes CtIP and MRE11 (Gaymes et al., 2013). Furthermore, Olaparib and Veliparib hypersensitivity has been demonstrated in patient-derived myeloproliferative neoplasms irrespective of BRCA1/2 mutational status. However, greater PARPi sensitivity was observed in samples which possessed a DNA damage repair defect (Pratz et al., 2016).

Several AML-inducing fusion proteins have been shown to confer PARPi sensitivity in cellular models. For instance, Olaparib has been shown to have significant additive effects on the anti-tumor activity of two chemotherapy drugs, Doxorubicin and Cytarabine, in MLL-AF9-positive mouse models (Stavropoulou et al., 2018). AML1-ETO and PML-RARα are well recognized AML-associated fusion proteins, shown to promote leukemogenesis (Singh et al., 2017). Esposito et al. (2015) demonstrated that AML1-ETO or PML-RARα positive models possessed PARPi sensitivity due to a jeopardized DDR and the down-regulation of HR genes, shown to be mediated by HOXA9 activity (Esposito et al., 2015). There are currently several clinical trials underway to investigate PARPi use in AML patients; however, the majority of these are still in the recruitment phase and results are not yet available.

## PARP1 Regulating Proteins as Potential New Biomarkers or Therapeutic Targets

Given the growing prevalence of PARPi resistance, it is essential that alternative PARP inhibiting mechanisms are investigated to improve treatment opportunities. Recent research has shown PARP1 activity is regulated by physical interactions with several other proteins, including HPF1, YB-1, Sam68, Banf1, TRIP12 and, as discussed earlier, PARG (Alemasova et al., 2016; Gibbs-Seymour et al., 2016; Sun et al., 2016; Gogola et al., 2018; Bolderson et al., 2019; Gatti et al., 2020). Therefore, modulation of these PARP1 regulatory proteins may provide an alternate method of downregulating PARP1 activity or modulating the sensitivity of tumor cells to PARP inhibitors.

Histone PARylation factor 1 (HPF1) has been shown to have an essential role in enabling the *trans* ADP-ribosylation of histones by PARP1 during the DNA damage response at serine residues (Gibbs-Seymour et al., 2016; Leidecker et al., 2016; Bonfiglio et al., 2017). HPF1 was also identified to be involved in the inhibition of PARP1 hyper-automotification induced by DNA damage, which may have a role in maintaining genomic stability (Gibbs-Seymour et al., 2016). Lastly, in *vitro* findings by Gibbs-Seymour et al. (2016) demonstrated that depletion of HPF1 induces sensitivity to PARPi treatment and other DNA damaging agents. Collectively, these findings suggest that HPF1 is involved in maintaining appropriate PARP1 activity, particularly by upregulating PARP1’s activity during the DNA damage response.

Furthermore, YB-1 (Y-box-binding protein) has also been shown to physically interact with PARP1 and PARP2 to promote the auto-PARylation of PARP and inhibit PARG-mediated PAR degradation (Alemasova et al., 2016). Subsequently, YB-1 was identified as a co-factor of PARP1 and shown to counteract the inhibition of PARylation induced by low dosages of PARPi in *vitro* (Alemasova et al., 2018). However, it was also shown that YB-1 was unable to entirely inhibit the effects of high dosages of PARPi (Alemasova et al., 2018). Together, these findings indicate that YB-1 plays a key role in the regulation of PARP1 activity via the regulation of PARP1/2 auto-PARylation.

Src-associated substrate during mitosis 68 kDa (Sam68) is a protein shown to localize at DNA lesions following damage. A physical interaction between Sam68 and PARP1 has been observed; however, similar interactions were not observed between Sam68 and PARP2, PARP3, PARP5a or PARP5b (Sun et al., 2016). Supporting its role as a positive regulator of PARP1 depletion of Sam68 in mice models resulted in impaired PARP1 activation, PAR chain development and activation of PAR dependent signaling, including the NF-κB pathway (Fu et al., 2016a, b). Sam68 depletion also resulted in similar phenotypes to those observed following PARP1 depletion (Sun et al., 2016). The role of Sam68 in PARPi sensitivity has not been examined to date. In summary, these findings suggest that Sam68 is a key regulator of PARP1 activation and subsequent downstream regulating.

We recently identified that Barrier to Autointegration Factor 1, Banf1 is a negative regulator of PARP1 activity (Bolderson et al., 2019). Banf1 was found to bind to the NAD^+^ binding domain of PARP1 and inhibit it’s auto-PARylation and activity toward histone substrates following oxidative stress. The role of Banf1 in the response of tumors to PARPi remains to be determined.

A recent study identified the ubiquitin E3 ligase TRIP12 as a regulator of PARP1 stability and PARPi-induced PARP trapping. As such, depletion of TRIP12 leads to an increase in PARPi-induced PARP trapping and induces replication stress, DNA damage and results in cell death. Hence, the levels of TRIP12 protein could be an important consideration for the sensitivity of tumor cells to PARPi (Gatti et al., 2020).

Given their role in the regulation of PARP stability and activity, modulation of HPF1, YB-1, Sam68, Banf1 and TRIP12 may provide novel combination therapies to potentiate the effect of existing PARP inhibitors or provide alternative targets for the development of new PARP inhibiting drugs. It is also possible that these regulators could act as biomarkers for the response of tumors to PARPi. However, the safety and efficiency of these targets in humans remains to be established.

## Conclusion

Since their discovery half a century ago, the PARP protein family has been proposed to have multiple functions in cellular processes; including transcription, cell death and DNA repair. In particular, knowledge of the basic biology and roles of PARP1 in DNA repair pathways led to the development of PARPi, for the targeted treatment of BRCA-mutated cancers. The potential of PARPi therapy in a variety of cancer subtypes has been highlighted by the significant numbers of preclinical studies and clinical trials, demonstrating their superior efficacy over traditional chemotherapies in some cancers. Studies have also established the substantial anti-tumor benefits of utilizing PARPi in combination with other anti-cancer agents to induce significant tumor regression. However, although the clinical relevance of PARPi is clear, the underlying mechanisms of PARPi activity remain elusive; therefore, limiting our understanding of potential targets for PARPi tumor biomarkers and pathways of therapy resistance. Further studies of the mechanism of action of PARPi are required, along with the validation and approval of additional biomarkers to ensure that PARPi therapy is utilized to provide maximal patient benefit.

## Author Contributions

All the authors contributed to writing and editing the manuscript. MR made the figure.

## Conflict of Interest

KO’B and DR are founders of CARP Pharmaceuticals. EB, DR, and KO’B are founders of Carpe Vitae Pharmaceuticals. EB, JB, KO’B, and DR are inventors on patent applications filed by Queensland University of Technology. The remaining author declares that the research was conducted in the absence of any commercial or financial relationships that could be construed as a potential conflict of interest.
